# A Highly Sensitive Giant Magnetoresistive (GMR) Biosensor Based on the Magnetic Flux Concentrator Effect

**DOI:** 10.3390/mi16050559

**Published:** 2025-05-03

**Authors:** Hao Sun, Jiao Li, Changhui Zhao, Chunming Ren, Tian Tian, Chong Lei, Xuecheng Sun

**Affiliations:** 1 Microelectronics Research & Development Center, School of Mechatronic Engineering and Automation, Shanghai University, Shanghai 200444, China; sun_hao@shu.edu.cn (H.S.); lijiaoshu@shu.edu.cn (J.L.); zhaochanghui2023@shu.edu.com (C.Z.); rcm@shu.edu.cn (C.R.); 1184388429@shu.edu.cn (T.T.); 2 National Key Laboratory of Advanced Micro and Nano Manufacture Technology, Department of Micro-Nano Electronics, School of Electronic Information and Electrical Engineering, Shanghai Jiao Tong University, Dongchuan Road 800, Shanghai 200240, China; leiqhd@sjtu.edu.cn; 3 Shanghai Key Laboratory of Automotive Intelligent Network Interaction Chip and System, The School of Microelectronics, Shanghai University, Shanghai 200444, China

**Keywords:** GMR sensor, biosensor, magnetic flux concentrator, *Staphylococcus aureus*

## Abstract

Magnetic biosensors have wide applications in biological target detection due to their advantages such as low background noise, convenient detection, and low requirements for sample pretreatment. However, existing magnetic biosensors still have the drawback of low sensitivity compared to optical and electrochemical biosensors. This paper presents the novel design of a high-sensitivity magnetic biosensor by utilizing the magnetic field line convergence effect, which was applied to bacterial detection. The results indicate that it can achieve a detection limitation of 10 CFU/mL, demonstrating that it can be implemented in high-sensitivity biological target detection.

## 1. Introduction

Biosensor technology is a new type of detection technology that specifically combines biomolecules with molecules that can be identified by the sensitive unit of a sensor. They transform the biological signal into a processable sensor signal, such as optical and electrical signals, after a biochemical reaction, so as to achieve the purpose of analysis and detection [1,2]. Biosensors, such as fluorescence sensors [3,4], electrochemical sensors [5,6,7], and magnetic sensors [8,9], demonstrate great sensitivity and efficiency in detecting biological samples. Nevertheless, certain sensors require complex preparation and have strict requirements for detection samples [10]. For instance, optical sensors need to be pretreated by centrifugation, filtration, and other methods to reduce noise [4,11,12]. Acoustic sensors also require pretreatment in complex matrices through pre-enrichment [13,14]. Magnetic nanoparticles can effectively separate and enrich target bacteria [15], which significantly improves detection sensitivity and specificity [16]. The giant magnetoresistive (GMR) sensor is a magnetic sensor used for biological detection. It combines immunomagnetic bead technology with the double-antibody sandwich method, which immobilizes biomolecules on the sensor surface [17]. The design lowers background noise and prevents interference from external non-magnetic sources [18,19]. The GMR sensor detects biomolecules by outputting a change in resistance signal, specifically the change in the magnetoresistance ratio (MR). Compared with other magnetic biosensors, GMR sensors have advantages such as simple processing, strong stability, high sensitivity, and ease of integration with Micro-Electro-Mechanical System (MEMS) technology [20]. They are currently widely used in various scenarios such as disease diagnosis, food safety, and environmental monitoring [21]. As a result, GMR magnetic sensor research and development are essential.

Conventional GMR sensors have limitations with regard to biological detection characteristics and their own properties. They have a single structural design (such as a strip and serpentine pattern) [22,23], and the shape of the magnetic film layer restricts the output signal’s linear response range. The sensitivity of these sensors is also limited by the sensitivity of the magnetic film [24,25]. Therefore, traditional GMR sensors still present challenges for the detection of biomolecules in low concentrations or complex matrices [26].

At present, GMR magnetic biosensors have made some progress in the detection of various biomolecules such as DNA [27,28,29], proteins [29], and bacteria [30]. However, the sensitivity of the sensor must be further enhanced in order to raise the limit for biomolecule detection. Since magnetic bioassays use immunomagnetic beads (IMBs) as a medium to capture the measured biomolecules (such as bacteria) [31], the GMR sensor needs to be highly sensitive for the detection of magnetic beads. The magnetic flux concentrator (MFC) is a device that can direct magnetic flux under a static magnetic field [32], and the magnetic flux surrounding the concentrator increases the sensitivity of the sensor [33]. The addition of an MFC to the GMR sensor can improve the limitation of detection, but current MFC devices are basically externally placed in the magnetic sensor.

In this work, a novel GMR sensor structure based on MFC was proposed and prepared by MEMS technology. To analyze the impact of concentrators on sensor and bead detection through simulation, the GMR sensor was measured using a testing platform to verify its stability and sensitivity. *Staphylococcus aureus* (*S. aureus*) samples were used to evaluate the performance of a GMR sensor based on a MFC for the detection of real biological targets for magnetic bead immunocapture.

## 2. Simulation and Analysis

### 2.1. Principle and Model of Magnetic Flux Concentrator

Magnetic flux concentrators (MFCs) are usually a pair of symmetrical structural devices [32], composed of high-magnetic-permeability materials such as iron, nickel, cobalt, etc., with gaps left in the middle. When the magnetic flux concentrator is in a uniform static external magnetic field *B_ext_*, a large number of surrounding magnetic flux lines will converge towards the concentrator, and the density of the magnetic flux lines in the air gap will increase significantly. The air gap magnetic field *B_g_* detected by the magnetic sensitive unit will also increase significantly. Therefore, magnetic flux concentrators can effectively improve the detection sensitivity of magnetic sensitive units. To characterize the amount of magnetic field growth caused by the guidance of magnetic flux, the gain of magnetically induction intensity in the air gap of the concentrator is defined as *G* [33]:

(1) $$G=\frac{B_{g}}{B_{ext}}$$

**Figure 1:**
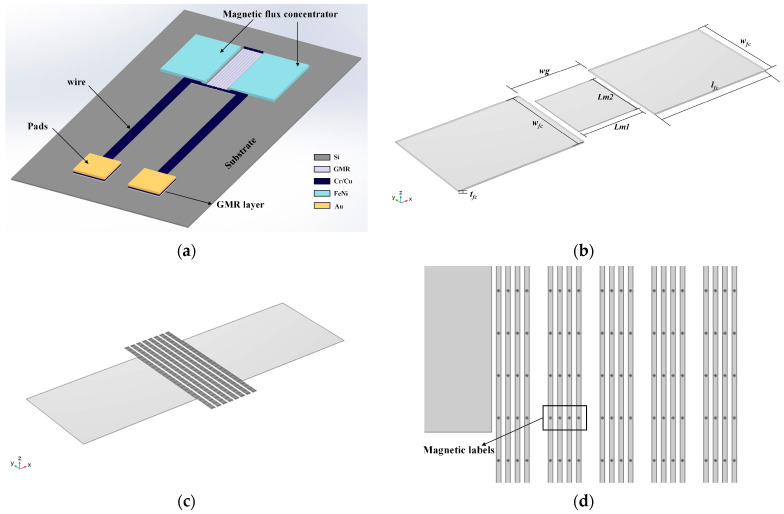
The schematic diagram of the GMR magnetic sensor based on MFC: (**a**) structural design of the GMR sensor; (**b**) initial model; (**c**) sensor model with a uniform distribution of magnetic beads; (**d**) details of the beads.

The finite element simulation software COMSOL Multiphysics 6.0 was used to establish the GMR sensor model of the integrated magnetic flux concentrator. Ni was selected as the material of the MFC, and the relative permeability μr was 1000. The magnetic field distribution without a conduction current was simulated and calculated using the Finite Element Method (FEM). Figure 1b shows the model of the GMR magnetic sensor based on an MFC, which is mainly composed of the GMR sensitive unit and the MFC.

The primary structural parameters of the GMR magnetic sensor model are displayed in Table 1. The size of the sensitive area for middle detection is 500 μm × 500 μm × 2 μm. The MFC is symmetrically placed around the middle detection area and is a rectangular model of 1000 μm × 750 μm × 2 μm. The air gap width w_g_ between the two MFCs is 600 μm.

The GMR detection sensitive region in this research was equal to a thin rectangular structure in order to investigate the impact of the MFC on the sensitivity of the GMR magnetic sensor. Under the *B_ext_* of the external magnetic field of 0.001 T, the width of the sensitive region and the width of the air gap were simulated and calculated, and their effects on the sensitivity were obtained.

Further study of the influence of the MFC-based sensor on the actual detection of the magnetic label was conducted by using the magnetic bead model simulation calculation. Additionally, we investigated how the distribution of the magnetic beads affects the magnetic field in the sensitive area of the GMR sensor. Figure 1c displays a schematic diagram of the sensor model with a uniform distribution of magnetic beads. The sensitive area was modified to a stripe shape to simulate the actual GMR stripe, and the magnetic beads were evenly distributed on the surface. The air gap width w_g_ was 510 μm, and the sensitive area width Lm2 was 1100 μm. The sensitive area was set to 32 thin cuboids to simulate 8 groups of GMR stripes in series. Each group was composed of 4 thin cuboids of 5 μm × 1100 μm × 2 μm, with a spacing of 5 μm. The magnetic beads in the model were set as spheres with a radius of 1.4 μm, and the material was set as soft ferromagnetic material. The spherical magnetic beads were evenly distributed on eight groups of strip cuboids, as shown in Figure 1d. The magnetic beads in each group were in a 4 × 25 array structure. The spacing between the magnetic beads in the x direction was 10 μm, and the spacing in the y direction was 45 μm.

### 2.2. Simulation Results and Analysis of MFC

#### 2.2.1. Sensitive Area Width Lm2

**Figure 2:**
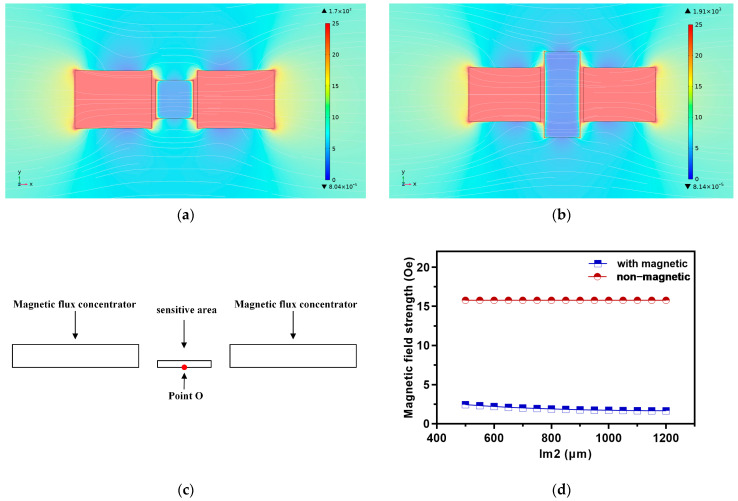

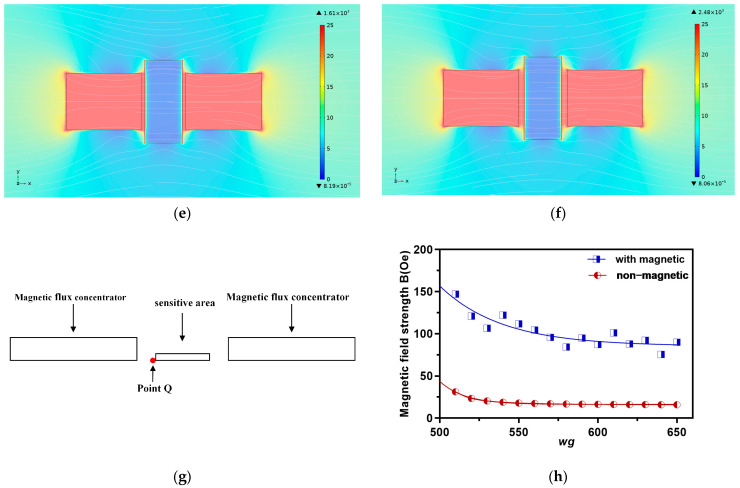
Simulation results of magnetic flux concentrators: (**a**) magnetic flux intensity distribution of Lm2 = 500 μm; (**b**) Lm2 = 1200 μm; (**c**) schematic diagram of the O point; (**d**) the effect of Lm2 on magnetic induction intensity; (**e**) magnetic flux density distribution of w_g_ = 510; (**f**) w_g_ = 650; (**g**) schematic diagram of the O point; (**h**) the effect of w_g_ on magnetic induction intensity at point Q.

We parameterized the width Lm2 of the sensitive area in the middle of the model in Figure 1b, ranging from 500 μm to 1200 μm, with a step size of 50 μm and Lm1 unchanged. Figure 2a,b displays the magnetic flux distribution and magnetic field strength simulation results with an Lm2 of 850 μm and 1200 μm. It was discovered that as the size of the sensitive area increases, the high magnetic field area of the concentrator spreads outwards, and the magnetic field distribution becomes more uniform, but the concentration decreases. Magnetic leakage increased as a result of the magnetic flux extending towards the edge. Additionally, there was a great dispersion of the magnetic flux in the sensitive region.

Due to the uniform distribution of the magnetic field in the sensitive area, the influence of the width Lm2 on the magnetic field at the center O point (0, 0, −1) of the unit was analyzed. It covered both magnetic and non-magnetic states in the sensitive area. As shown in Figure 2c,d, when the sensitive area was non-magnetic, the MFC concentrated the magnetic flux at the center O point of the detection area and there was very little change (less than 0.002 Oe) in the magnetic induction intensity measured at the same position O point. When the sensitive area was magnetic, the MFC diverted the magnetic field, significantly reducing the magnetic induction intensity. As the width Lm2 of the sensitive area increased, the magnetic induction intensity gradually decreased, and the magnitude of the change was larger than that of non-magnetic areas (between 1.6 Oe and 2.5 Oe).

#### 2.2.2. Air Gap Width w_g_

Due to the close correlation between the degree of magnetic field concentration in the detection area and the air gap width of MFC [34], further simulation calculation and analysis of the air gap width w_g_ were carried out to optimize the design of the MFC. The air gap contains a sensitive area in the middle, so the width of the air gap should be at least larger than the length of the sensitive area. The parameterized scanning of the air gap width w_g_ ranged from 510 μm to 650 μm, with a step size of 10 μm.

The magnetic flux density distribution in the sensitive area is shown in Figure 2e,f. When the w_g_ was very small, the magnetic induction intensity on both sides of the sensitive area was significantly amplified. The magnetic flux in the middle of the sensitive area was more concentrated, effectively improving the detection sensitivity. After the increase in w_g_, the distribution of magnetic flux became more uniform. Leakage magnetic flux was generated in all directions when the magnetic flux density decreased. The edge magnetic induction intensity in the sensitive area decreased as the air gap increased, according to the calculation results of the detecting edge point Q (−260, 0, −1) in Figure 2g,h. When the sensitive area was non-magnetic, the intensity of the edge magnetic field varied more steadily. In contrast, the edge magnetic field intensity shakes during a significant decrease.

#### 2.2.3. Magnetic Labels

The application of GMR magnetic biosensors mainly involves combining magnetic labels with targets, transmitting biological signals into magnetic signals, and finally converting the magnetic signals into electrical signals. Therefore, simulation studies were conducted by adding magnetic labels to sensitive areas. We calculated and analyzed two cases: one in which the sensitive area is a single sheet-like magnetic label, and the other in which the sensitive area surface simulates the distribution of magnetic beads.

**Figure 3:**
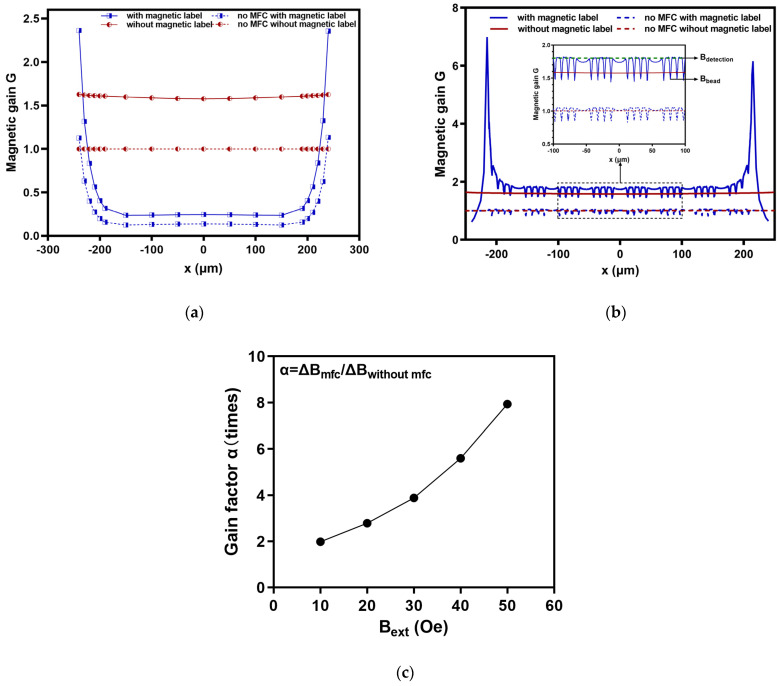
Simulation results with or without MFC for magnetic labels: (**a**) magnetic gain with or without sheet magnetic labels; (**b**) magnetic gain for uniform bead distribution; (**c**) the gain factor α under different exciting magnetic fields.

Following completion of the simulation of a single sheet-like magnetic label in the sensitive area, the magnetic gain of some points on the *x*-axis of the sensitive area was calculated according to Formula (1). The outcome is displayed in Figure 3a. It was found that when there was no magnetic label and the magnetic gain was stable, consistent with the phenomenon of magnetic field lines being concentrated in the detection area. When there was a magnetic label, the magnetic gain changed significantly. The magnetic flux in the detection area was evenly distributed, and the magnetic flux at the edges was diverted. In the stable region of magnetic gain, where the x-coordinate ranged from −200 μm to 200 μm, the difference in magnetic gain of the model with MFC was about 1.4. This means that in both cases, with and without magnetic labels, the sum of the rate of change in the magnetic field strength in the detection area relative to the external magnetic field Bext was approximately 140%. The difference in the magnetic gain of the model without MFC was approximately 0.86. By comparing the differences in the magnetic gain between models with and without an MFC, it can be demonstrated that an MFC can amplify the detection signals of magnetic labels. Therefore, sensors that integrate an MFC can achieve a highly sensitive detection of magnetic labels.

After the simulation of the model in Figure 1c, the magnetic gain of the sensitive area on the *x*-axis was calculated in the same way to obtain the calculation results in Figure 3b. The magnetic shunt was obvious in the distribution of magnetic beads and the magnetic gain G was smaller than that of the strip sensitive area without magnetic beads, showing regular changes. Due to the stray magnetic field generated by the magnetic beads themselves, the magnetic gain in the middle part of the strip-like sensitive area was doubled compared to the single sheet-like magnetic label. As the research goal, we considered a fifth group of uniformly distributed magnetic beads on the *x*-axis. It was found that the surface G on the sensitive area without magnetic bead distribution was about 1.8, which means that the magnetic induction intensity increased by 80% compared to the external magnetic field *B_ext_*. The magnetic gain of the position with beads decreased to 1.45–1.48, indicating an increase in magnetic induction intensity of 45–48%. Although the magnetic beads have a weakening effect on the local magnetic field, this can still prove that the detection signal intensity with magnetic bead distribution on the sensor surface increased. By calculating the magnetic gain difference ΔG between the magnetic bead distribution area and the adjacent bead-free area, it was found that the relative magnetic gain rate of the magnetic bead distribution area was about 30%. In the model without MFC, the surface G in the region without magnetic bead distribution was about 1, meaning that the magnetic induction intensity was the same as the external magnetic field *B_ext_*. The magnetic gain at the position of the beads was 0.87 to 0.91, indicating a reduction in the magnetic induction intensity of 9% to 13%. After calculating the magnetic gain difference, it was discovered that the distribution area of the beads had a relative magnetic gain change rate of about 12%.

The detection signal of a GMR sensor for bacteria is determined by the weakening effect induced by the magnetic beads. The weakening effect is the difference between the magnetic field at the GMR sensor detection area (*B_detection_*) and the stray fields originating from magnetized beads (*B_bead_*), as shown in Figure 3b. Here, the difference in the weakening effect was defined as Δ*B*:

(2) $$\Delta B=B_{detection}-B_{bead}$$

The weakening effect with MFC as Δ*B_mfc_*, the weakening effect without MFC as Δ*B_without mfc_*, and the gain effect was defined as *α*:

(3) $$\alpha =\frac{\Delta B_{mfc}}{\Delta B_{without\;mfc}}$$

Based on the data in Figure 3b, *α* was calculated as 1.98 when *B_ext_* was 10 Oe, which means the weakening effect of magnetic bead with the MFC was 1.98 times that without an MFC. The α at 20 Oe, 30 Oe, 40 Oe, and 50 Oe was also calculated, as shown in Figure 3c, which confirmed that the gain effect was clearly enhanced when the exciting magnetic field increased. Thus, GMR sensors based on an MFC effectively improve the sensitivity of magnetic bead detection.

## 3. Experimental Details

### 3.1. Design and Fabrication of GMR Magnetic Sensor

**Figure 4:**
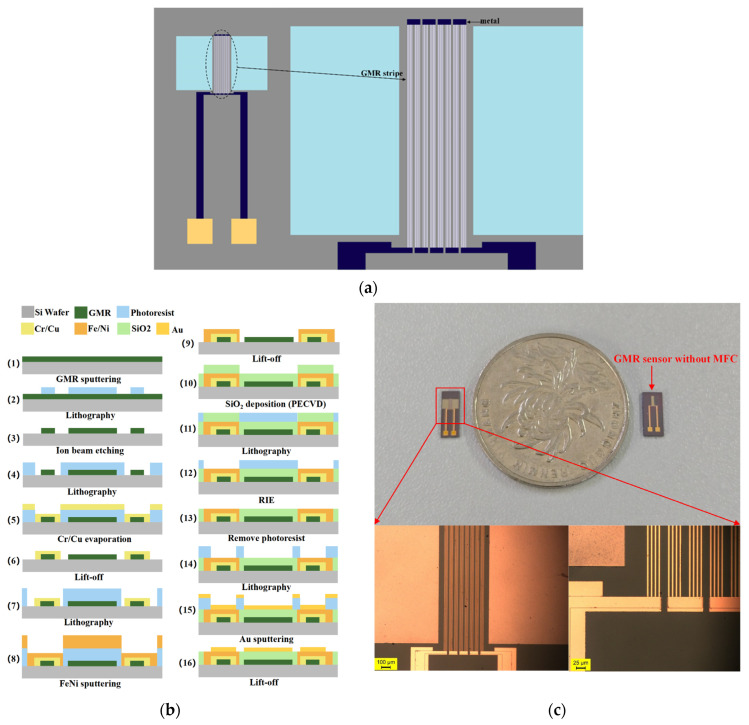
Design and fabrication of GMR magnetic sensor: (**a**) top view of structural design; (**b**) the manufacturing process of the GMR sensor; (**c**) the fabricated GMR sensor.

The GMR magnetic sensor has a multi-layer structure, mainly consisting of the substrate layer, GMR stripe layer, wire pin layer, magnetic flux concentrator layer, and gold film layer. The structural design is shown in Figure 4a. The structure design of the GMR stripes was different from in other studies, with a line width of 5 μm, spacing of 5 μm, length of 1600 μm, 4 continuous turns, and a parallel connection as a group. There were a total of 8 sets of GMR stripes connected in series, with a spacing of 20 μm. The magnetic flux concentrator was integrated into the GMR sensor through a FeNi thin film. The size of the magnetic flux concentrator was 1000 μm × 1500 μm, and the distance between the upper end of the concentrator and the GMR was 20 μm while the lower end was 80 μm.

The processing and manufacturing of GMR magnetic sensors are mainly achieved through micro-nano processing technology. GMR thin film sputtering, photolithography, and ion beam etching were used to pattern the GMR thin film to obtain GMR stripes. The conductive layer of the GMR sensor was prepared by Cr/Cu sputtering, lift-off, and other processes. The protective film SiO_2_ was prepared by PECVD, and then the Au layer was prepared as a biochemical reaction layer and conductive electrode for subsequent testing and packaging. The specific fabrication process is shown in Figure 4b, and the steps were as follows.

The first step was the preparation of GMR film: GMR spin valve films were sputtered on Si/SiO_2_ substrates by a multi-target magnetron sputtering machine. The spin valve film structure was pinned on the bottom layer of Si/Ta (5)/FeNi (2)/IrMn (8)/CoFe (2)/Ru (0.8)/CoFe (2)/Cu (2.3)/CoFe (1.5)/FeNi (2)/Ta (3); (GMR film was prepared by Jiangsu Duowei Technology Co., Ltd., Changzhou, Jiangsu, China).

The second step was GMR stripe graphing: First, 5 μm positive glue was spun on the surface of the GMR wafer, the pattern of the corresponding mask (opaque) was exposed and developed, and then it was cleaned with deionized water and dried with N_2_. Then, the GMR stripes were etched by ion beam for 15 min. Finally, it was soaked in acetone for 2–3 h, and then cleaned with deionized water and dried at 90 °C for 2 h.

The third step was to prepare the wire electrode layer: The wire electrode layer of the sensor was prepared by the lift-off process. First, 5 μm positive glue was spun on the substrate; the second layer of the mask plate (transparent) was used for photolithography, developed, and then the electrode and wire area were exposed. Then, the Cr/Cu layer (10 nm/150 nm) was evaporated on the whole device surface to complete the coating of the wire and electrode layer. Finally, the device was immersed in acetone solution for 30 min to remove the excess photoresist on the surface, washed it with alcohol and ionic water 2–3 times, dried with N_2_, and dried it in an oven at 60 °C for 30 min.

The fourth step was to prepare the magnetic flux concentrator: The magnetron sputtering machine was used to sputter the magnetic thin film FeNi. The lift-off process was used to remove the glue, followed by cleaning and drying to complete the preparation of the thin film layer of the MFC.

The fifth step was the preparation of a protective layer: SiO_2_ thin films of 200 nm were deposited on the previously constructed device by plasma-enhanced chemical vapor deposition (PECVD). Then, 5 μm of positive glue was thrown on the surface, aligned with the fourth layer of mask plate (transparent), followed by photolithography, development, and exposing the electrode area. Then, reactive ion etching (RIE) was used to etch the SiO_2_ film on the electrode surface for 200 nm until all the electrodes of the wire layer were exposed. Finally, the glue was removed with acetone, the device was washed with alcohol 2–3 times, and then it was washed with deionized water 2–3 times, blow dried with N_2_, and dried in the oven at high temperature.

The sixth step was the preparation of gold film: The Au film was sputtered at 150 nm by a magnetron sputtering machine, and the photoresist with Au film on the surface was removed by lift-off to complete the preparation of the gold film. Finally, it was soaked in ionic water, washed 2–3 times, blow dried with N_2_, and put it in the oven for 2 h to dry.

### 3.2. Preparation of Biological Samples

#### 3.2.1. Biological and Chemical Materials

*Staphylococcus aureus* (*S. aureus*) is a common foodborne pathogen with a short incubation period [35]. It can enter food consumed by humans through multiple routes and may cause food poisoning within minutes to hours after entering the intestinal tract [36]. Given that *S. aureus* poses a significant threat to human health and food safety, it is essential to conduct highly sensitive testing for *Staphylococcus aureus*.

*S. aureus*, *S. aureus* monoclonal antibodies, and biotinylated *S. aureus* polyclonal antibodies were procured from Lianzu Biotechnology Co., Ltd. (Shanghai, China). *Shigella*, *Escherichia coli*, *Brucella*, and *Baumannii* were also procured from Lianzu Biotechnology Co., Ltd. (Shanghai, China). Dynabeads^TM^ M-280 Streptavidin and N-Hydroxysuccinimide (NHS) were sourced from Thermo Scientific^TM^ (USA). Phosphate-buffered saline (PBS) (pH = 7.4), 11-mercaptoundecanoic acid (11-MUA), 1-ethyl-3-(3-dimethylaminopropyl) carbodiimide (EDC), and bovine serum albumin (BSA) were obtained from Yuanye Biotechnology Co., Ltd. (Shanghai, China). Acetone was purchased from Sinopharm Chemical Reagent (Shanghai, China). The water used for cleaning in all experiments was deionized water.

#### 3.2.2. Preparation of Bacterial Samples

When using GMR magnetic sensors for biological detection, the dual-antibody sandwich method and immunomagnetic bead technology are used to convert biological signals into magnetic signals from magnetic nanoparticles [25,37]. Therefore, it is necessary to separately prepare magnetic bead immunocaptured bacteria and surface functionalized GMR biosensors. 

**Figure 5:**
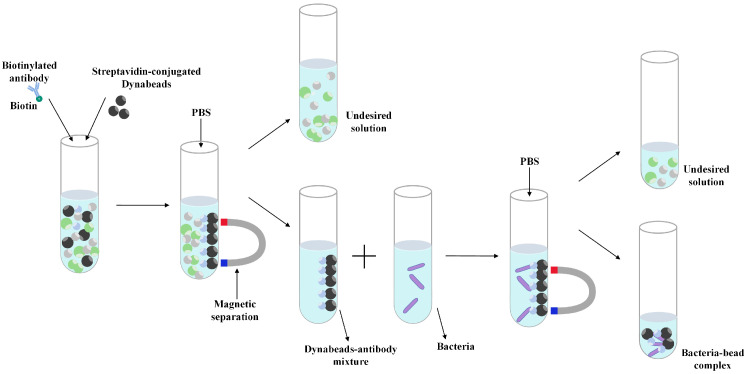
Schematic diagram of magnetic concentration.

The IMB solution of *S. aureus* antibody was prepared by mixing 100 µL of biotinylated *S. aureus* polyclonal antibody solution (0.1 mg/mL) with 100 µL of streptavidin magnetic bead solution (0.1 mg/mL). The mixed solution was incubated at 37 °C for 30 min to achieve the binding of magnetic beads and bacterial antibodies. Unbound antibody and other impurities were removed by magnetic separation, and the schematic diagram of magnetic separation is shown in Figure 5.

Five different concentrations of *S. aureus* samples (10 CFU/mL, 25 CFU/mL, 50 CFU/mL, 100 CFU/mL) were prepared in PBS solution. The antibody IMB solution was mixed with five concentrations of bacteria, respectively, and incubated at 37 °C for 30 min so that the antibody on the magnetic beads captured the bacteria. Once again, unbound bacteria and other impurities were removed through magnetic separation to complete the preparation of bacterial samples for magnetic bead immune capture.

#### 3.2.3. Surface Functionalization Modification

The surface of the GMR sensor was modified by chemical self-assembly monolayer technology based on bio-probes [38,39]. First, the chip was cleaned with acetone and alcohol 2–5 times, and then cleaned with deionized water 2–5 times and dried with N_2_. Then, the GMR sensor was soaked in 30 mmol/L 11-MUA solution and self-assembled for 3 h at room temperature. Then, 20 mL of 0.2 mol/L EDC and 20 mL of 50 mmol/L NHS solution were mixed, and the GMR sensor was soaked in the solution and activated at room temperature for 40 min. The monoclonal antibody against *S. aureus* was prepared in PBS (pH 7.4) at a concentration of 0.5 to 1.0 mg/mL. Then, 5 μL of antibody solution was dropped on the Au film on the sensor surface and incubated at 37 °C for 30 min. After that, the sensor was rinsed twice with PBS containing 1% BSA (pH 7.4). Finally, 10 μL of BSA (1% BSA, 0.2% Tween 20) solution was used to block the nonspecific binding sites on the surface of GMR, which was incubated for 1 h at room temperature. The sensor was washed with PBS (pH 7.4) 2–5 times and dried at room temperature.

## 4. Results and Discussion

### 4.1. Measurement of GMR Basic Performance

**Figure 6:**
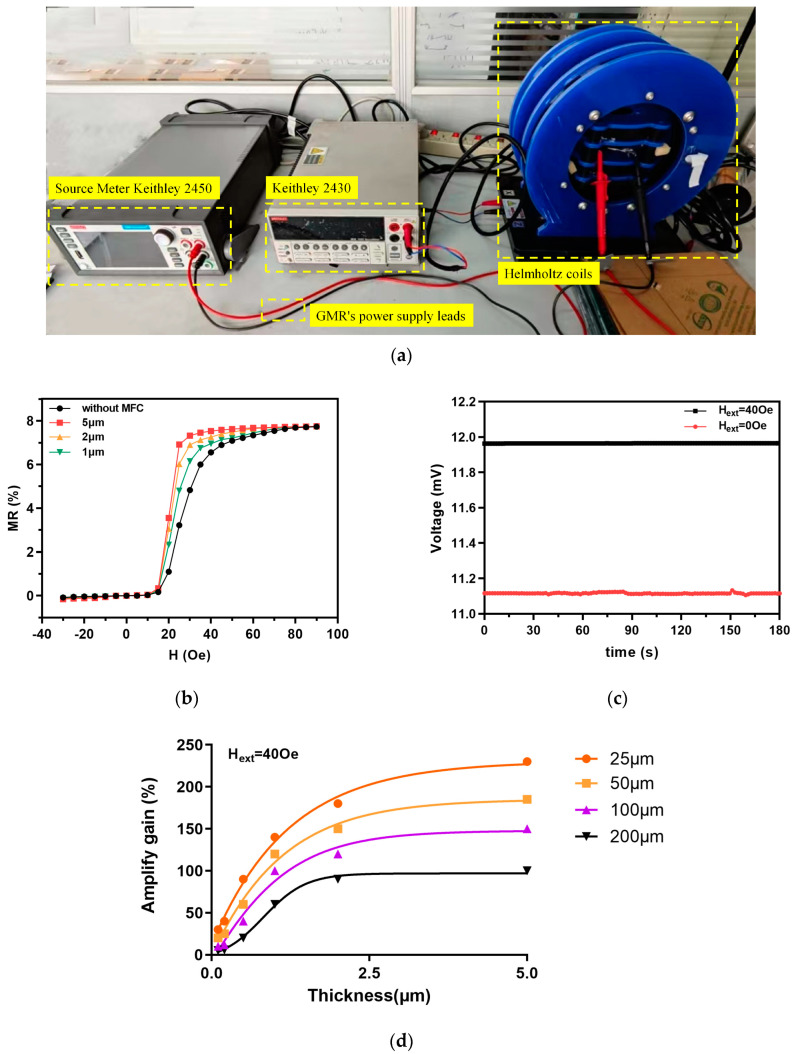
The measurement of the GMR magnetic sensor: (**a**) testing platform; (**b**) the basic curve of the GMR sensor (MR–H) with different thicknesses; (**c**) the stability curve of the GMR sensor; (**d**) amplified gain of the GMR sensor output by various MFCs.

The fabricated GMR magnetic sensor is shown in Figure 4c. The testing platform, as shown in Figure 6a, conducted preliminary performance tests on the GMR magnetic sensor. The Keithley2450 injected excitation current in the GMR sensor and measured the output voltage change in the sensor. The Keithle2430 provides direct current power to the Helmholtz coil, generating a magnetic field in the x-direction and controlling the magnitude of the magnetic field generated by the Helmholtz coil.

The detection principle of a GMR sensor is that the sensitive area of the GMR is affected by an external magnetic field, resulting in a change in magnetic resistance. The magnetic resistance change rate is the MR:

(4) $$MR=\left(\frac{R_{H}-R_{0}}{R_{0}}\right)\times 100$$

where *R_H_* is the sensor resistance under the external magnetic field exciting, and *R*_0_ is the sensor basic resistance without the external magnetic field exciting.

The complete MR–H basic performance curve obtained from the test is shown in Figure 6b. All curves represent the increase in the external magnetic field from 0 Oe to 90 Oe and the decrease from the reverse saturation magnetic field −30 Oe to 0 Oe. The fundamental MR of the GMR sensor utilized in the studies that follow in this paper is represented by the red curve. Initial performance testing of the GMR sensor showed that the test unit had a basic resistance value of 11.1 kΩ and a maximum MR of 7.75%. The sensitive region of the sensor reacted violently and exhibited significant changes in magnetic resistance when exposed to an external magnetic field of 20 Oe. The magnetic sensitivity within the range of 15–25 Oe was 0.71%/Oe. The black curve is the MR of the GMR sensor without an MFC, and the maximum MR was 7.73%. In the 15–25 Oe range, the magnetic sensitivity was 0.30%/Oe, and the magnetic resistance changed slowly. This proves that the MFC improved the sensitivity of the GMR sensor by 2.37 times, laying a foundation for subsequent sensitivity improvement in biological target detection. As the thickness decreases, the magnetic sensitivity in the range of 15–25 Oe also gradually declines. The MFC increased the magnetic sensitivity within a narrower magnetic field range by speeding up its reaction to the magnetic field. This means that the sensitivity of biological target detection may be improved, which could be tested by biological experiments.

The stability of the GMR magnetic sensor at geomagnetic fields of (0 Oe) and 40 Oe was tested separately. An excitation current of 1 μA was applied to the GMR sensor using a digital source meter and the amplitude range of the voltage output signal of the GMR sensor was detected within 3 min. Figure 6c reveals that the output voltage is generally stable when no external magnetic field is applied. However, the output signal may jitter due to the ambient magnetic field, and the variation range is within 0.4 mV. When a nearly saturated magnetic field of 40 Oe is applied, the output signal of the sensor is more stable, with a jitter range within 0.005 mV. In summary, the GMR sensor has a certain degree of stability during detection. Under the action of a stable external magnetic field, the output signal will be more stable. Therefore, when the GMR sensor is used for magnetic biological detection, the MR is reliable as the output.

To better analyze the effect of the MFC on the magnetic gain of the GMR sensor, we fabricated various MFCs with different thicknesses and spacings using the same process. We then measured the output *MR_mfc_* of the GMR sensor with the MFC at 40 Oe. Compared with the output *MR_non-mfc_* of the GMR sensor without an MFC, the amplify gain was calculated as follows:

(5) $$Amplify\;gain=\left(\frac{MR_{mfc}}{MR_{non-mfc}}-1\right)\times 100$$

The calculation results are shown in Figure 6d, where the gain increases with the thickness of the MFC. Additionally, the magnetic gain significantly increased with the decreasing distance between the MFC and the GMR sensitive region. Therefore, it was confirmed that the MFC can effectively enhance the sensitivity of the GMR sensor and amplify the output signal.

### 4.2. Magnetic Biological Detection

This study evaluated the magnetic biosensing performance of a GMR sensor using samples of *S. aureus*. The basic detection theory involved a magnetic dipole induced in the magnetic Dynabeads under the magnetization of the DC magnetic field. This inductive weak magnetic field is generally monodirectional and opposite to the DC magnetic field. As a result, the presence of magnetic beads causes a decrease in the effective DC magnetic field experienced by the GMR sensor. Thus, beads can be detected based on the change in the measured magnetic field measured by the sensor.

The basic operation is as follows: 5 µL of the prepared IMB–multi-antibody–*S. aureus* conjugate sample was taken and dropped onto the surface of the sensor’s sensitive area. Following incubation for 30 min, after the immune reaction was captured on the surface, the sensor was washed with PBS and dried. Finally, an external magnetic field was applied on the testing platform to complete the detection of the GMR biosensor. The output signal of the GMR sensor for detecting bacteria was defined as Δ*MR*:

(6) $$\Delta MR=MR_{sample}-MR_{initial}$$

*MR_initial_* is the initial magnetic resistance ratio without surface modification, while *MR_sample_* is the magnetic resistance ratio after surface immune capture of target bacteria.

**Figure 7:**
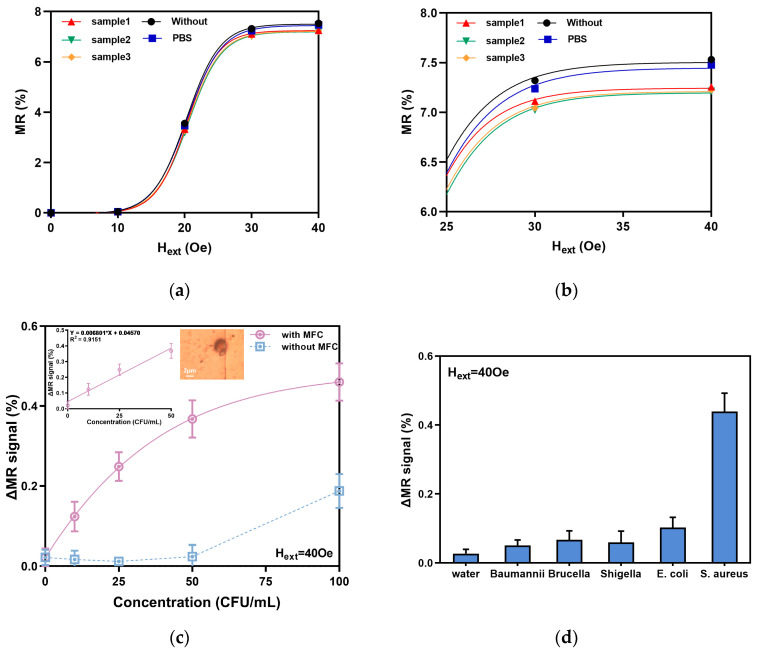
Bacteria detection results of the GMR sensor: (**a**) the relationship between the MR and H_ext_; (**b**) the amplification of the MR and H_ext_ relationship at 25–30 Oe; (**c**) the relationship between the output of GMR (ΔMR) and *S. aureus* in different concentrations; (**d**) specific test for bacteria at 50 CFU/mL.

The GMR magnetic sensor conducted repetitive testing on samples with a single concentration of 10 CFU/mL, and comparisons were made with a blank group and a PBS group, as illustrated in Figure 7a. The MR curves of the bacterial samples align with the basic performance curve. The MR values of the magnetic resistance ratio are similar under low magnetic fields. Meanwhile, at near-saturation magnetic fields (40 Oe), the MR value of the control group is greater than that of the bacterial group. The MR deviation for the same concentration of bacteria tested was less than 0.044%.

Further analysis of Figure 7b reveals that the signal exhibits greater distinguishability in the range of 30–40 Oe, rather than within the maximum sensitivity range (15–25 Oe). This is primarily due to the magnetic field generated by the magnetic beads, which opposes the applied excitation magnetic field, thus diminishing the effect of the applied magnetic field on the sensor. As the external magnetic field increases, the weakening effect becomes more apparent. This phenomenon is also observed beyond the 40 Oe magnetic field. Therefore, we ultimately selected a 40 Oe magnetic field as the excitation magnetic field to detect different concentrations of *S. aureus*.

Currently, existing GMR sensors lack the capability to detect bacteria at low concentration ranges [40]. Herein, we employed both GMR sensors with and without an MFC to test for bacteria at low concentration ranges. As shown in Figure 7c, the ΔMR signals of *S. aureus* samples at different concentrations (0 CFU/mL, 10 CFU/mL, 25 CFU/mL, 50 CFU/mL, and 100 CFU/mL) are presented. The GMR sensor without the MFC could not detect *S. aureus* below 100 CFU/mL, and the measured ΔMR signal was similar to that of the non-bacterial sample. In contrast, the GMR sensor with the MFC showed significant differences in ΔMR in the sensitive areas, corresponding to different concentrations of *S. aureus*. Therefore, it can clearly distinguish different concentrations of *S. aureus* samples with good sensitivity. The fitting equation is Y = 0.006801 × X + 0.04570, and the correlation coefficient R^2^ = 0.9151. The linearity is good in the low-concentration area, and the limit of detection was 10 CFU/mL for 10 strains of *S. aureus*. The sensitivity of bacterial detection with the MFC was 10 times higher than that without the MFC, and it was also 10 times higher than that of current conventional GMR sensors [30]. This mainly resulted from two factors: the first one is that the sensitivity of the GMR sensor was enhanced by the MFC, which improved the detection resolution of magnetic beads by the GMR sensor. The second one is that, under the same external magnetic field excitation, the magnetic field intensity of the GMR biosensor detection area with the MFC was stronger than that of the sensor without the MFC, making the weak magnetic field induced by the magnetic beads captured on the surface of the GMR biosensor with MFC stronger than that without the MFC, which improved the detection resolution of magnetic beads. Therefore, the detection limits for biological targets of the GMR sensor with the MFC was improved.

This work further conducted specificity testing on *S. aureus*, using four pathogenic bacteria, including *Brucella*, *Shigella*, *Escherichia coli*, and *Baumannni*, as non-specific target biomolecules. The specific detection was conducted under an external magnetic field of 40 Oe. The detection results are shown in Figure 7d, where *S. aureus* was accurately detected, while no obvious positive signals were detected for the other four bacteria. This further confirms that the GMR magnetic sensor has good specificity for analyzing the detection results of immunocaptured bacteria.

The detection results of the GMR sensor in this work were compared with other methods, as shown in Table 2. Currently, the GMR sensor has a detectable range for bacteria of 10^2^ to 10^3^, demonstrating good performance in detecting high concentrations of bacteria [30]. This confirms the applicability of the GMR sensor for high-concentration bacterial detection. However, there remains a gap in the detection of low-concentration bacteria [40]. Thus, we focus on the detection of bacterial samples below 100 CFU/mL. Compared to the GMR sensor without an MFC ([30] and this study), whose limit of detection was 100 CFU/mL, the minimum detection threshold for bacteria increased tenfold. In comparison with highly sensitive optical and electrochemical sensors [41,42,43], the detection limit of the GMR sensor proposed in this paper was on par with them, achieving a high level of sensitivity.

## 5. Conclusions

In summary, a novel GMR magnetic biosensor based on an MFC was introduced. The simulation experiment results clearly indicate that the sensitivity of the sensor with the MFC was feasibly improved. The magnetic gain change rate of the MFC for detecting the presence or absence of magnetic labels reached 30%. We optimized the structural parameters of the sensitive area based on the simulation results. GMR magnetic sensors with an integrated MFC were fabricated by using MEMS technology. The magnetic resistance ratio, MR, of the GMR sensor was 7.75%, and the magnetic sensitivity in the range of 15–25 Oe was 0.71%/Oe. The results indicate that the sensor has advantages such as low background noise, high stability, miniaturization, and high sensitivity. Due to the immunomagnetic beads (IMBs) that were implemented for bacterial target capture and detection labeling, as well as magnetic concentration of samples, the limit of detection was up to 10 CFU/mL. The detection result signals for different bacterial species was negative, indicating that the sensor can detect specific bacteria. In the future, we aim to apply this high-sensitivity sensor to array sensor chips to achieve high-throughput detection of multiple types and concentrations of target bacteria.

## Figures and Tables

**Table 1:** Structural parameters for the design of the GMR model with magnetic flux concentrator.

Parameter	Describe
Lm1	The length of the magnetic sensitive region
Lm2	Width of magnetic sensitive region
l_fc_	Length of MFC
w_fc_	Width of MFC
t_fc_	Thickness of MFC
w_g_	Width of air gap

**Table 2:** Comparison of our GMR sensor with an MFC with other biosensor methods.

Reference	Year	Linear Range (CFU/mL)	LOD (CFU/mL)	Methods	Bacteria
[30]	2016	10^2^–10^3^	100.0	Magnetoresistance	*E. coli*
[41]	2021	6.0 × 10^1^–6.0 × 10^7^	9.0	Electrochemical (DPV)	*S. aureus*
[42]	2022	10^1^–10^7^	29.0	Fluorescence	*S. aureus*
[43]	2023	2.0 × 10^1^–10^8^	7.0	Fluorescence	*S. aureus*
This study	2025	10^1^–10^2^	10.0	Magnetoresistance	*S. aureus*

## Data Availability

The original contributions presented in the study are included in the article; further inquiries can be directed to the corresponding author.
